# Correlates of illicit methadone use in New York City: A cross-sectional study

**DOI:** 10.1186/1471-2458-8-375

**Published:** 2008-10-28

**Authors:** Danielle C Ompad, Crystal M Fuller, Christina A Chan, Victoria Frye, David Vlahov, Sandro Galea

**Affiliations:** 1 Center for Urban Epidemiologic Studies, New York Academy of Medicine, New York, USA; 2 Mailman School of Public Health, Columbia University, New York, USA; 3 University of Michigan School of Public Health, Ann Arbor, Michigan, USA

## Abstract

**Background:**

Despite growing concern about illicit methadone use in the US and other countries, there is little data about the prevalence and correlates of methadone use in large urban areas. We assessed the prevalence and examined correlates of lifetime and recent illicit methadone use in New York City (NYC).

**Methods:**

1,415 heroin, crack, and cocaine users aged 15–40 years were recruited in NYC between 2000 and 2004 to complete interviewer-administered questionnaires.

**Results:**

In multivariable logistic regression, non-injection drug users who used illicit methadone were more likely to be heroin dependent, less than daily methamphetamine users and to have a heroin using sex partner in the last two months. Injection drug users who used illicit methadone were more likely to use heroin daily, share injection paraphernalia and less likely to have been in a detoxification program and to have not used marijuana in the last six months.

**Conclusion:**

The results overall suggest that illicit (or street) methadone use is likely not a primary drug of choice, but is instead more common in concert with other illicit drug use.

## Background

Methadone maintenance treatment programs (MMTP) have been shown to be effective for the management of opiate addiction, but remain controversial with respect to placement of clinics [1]. Between 1920 and 1964, physicians who treated substance users with opioids were subject to prosecution [2]. Among the many concerns of opponents of MMTPs, the diversion of methadone for illicit use is a key issue and has been described as "...perhaps the single greatest threat to the legitimate treatment of heroin addiction..." [3]. As a result, methadone programs are highly regulated by the U.S. government and practitioners are required to register with the U.S. Drug Enforcement Agency and be accredited by the Substance Abuse and Mental Health Services Administration [2,4]. In other countries, provision of methadone is not as restricted. Methadone use is not restricted to the treatment of heroin dependence, but is also used to manage pain.

There are few studies that have documented the prevalence and correlates of illicit methadone use. More than a decade ago Lauzon and colleagues [5] examined illicit methadone use among injection drug users (IDUs) in Montréal. They found that none reported methadone as their drug of choice, but the lifetime prevalence of use was 59.4% among those who preferred heroin and 26.7% among those who preferred cocaine. Mean age of initiation of methadone was older than the mean age of initiation of other substances in this study. More recently, one study in Australia examined methadone syrup injection among heroin users [6] and reported a lifetime prevalence of 18.4%, with 11% reporting use in the last six months (7.4% used diverted methadone). Methadone syrup injection was more prevalent among men.

Data about the behavioral correlates of illicit methadone use are sparse. An early study reported that most illicit methadone users did not initiate opiate use with methadone and that their use was sporadic [7]. More recently, a Montréal study found that illicit methadone use was more common about those who used heroin, either alone or in addition to cocaine, as compared to those who only used cocaine [5]. An Australian study that examined methadone syrup injection found that users were riskier than those who had not injected methadone; being more likely to have overdosed, used heroin by themselves and be a polysubstance user [6].

In the U.S. there has been particular concern about the increase in prescription drug-related deaths, and thus many studies assessing the impact of methadone have focused on methadone-related mortality [c.f., [8-10]]. Several recent studies have looked at overdose deaths and reported that between 2 and 34% of overdose deaths either involved methadone or were caused by methadone [11-14]. Reports have suggested increases in overdose deaths due to methadone [15,16]. However, few studies have specifically examined the role of illicit methadone in fatal overdoses; those that have estimate that between 13 and 57% of methadone-related deaths were due to illicit methadone [14,17-19]. In New York City (NYC) in particular, deaths attributable to methadone accounted for 13–16% of accidental overdose deaths between 1990–1998 [11]. However, this latter study was unable to determine if the decedents were in MMTP at the time of death or if the methadone had been illegally obtained. One study found that mortality related to drug poisoning was significantly correlated with drug sales for methadone and oxycodone [20]. In US studies, decedents were generally more likely to be male [14,18], White [14,18], and older adults [11,14,18].

Given the concern about illicit methadone use in the US and other countries and the rate of methadone-related overdoses in NYC, we sought to extend our previous work focusing on methadone-related mortality [11]. Here, we present data on the prevalence and correlates of illicit methadone use among both injection and non-injection drug users in NYC. It is the aim of this study to further understand the epidemiology of illicit methadone use in the United States. We hypothesized that illicit methadone use was associated with heroin dependence, and therefore the prevalence of use among non-injection drug users (NIDUs) would less than that of injection drug users (IDUs).

## Methods

### Subject recruitment

Potential participants were recruited to participate two ongoing studies of NIDUs and IDUs [21,22] using "street outreach" techniques, as described elsewhere [23,24]. Briefly, outreach workers engaged drug users in conversations about ongoing research at the research storefronts or a mobile van parked in communities where drugs were bought and/or used. We recruited participants from three NYC boroughs including: Harlem, the South Bronx, and the Lower East Side in Manhattan; Jamaica and Queensbridge in Queens; and Bedford-Stuyvesant in Brooklyn. Both IDUs and NIDUs were recruited into two cohort studies between 2000 and 2004. Potential participants completed a screening demographic interview. The IDU study was designed to investigate correlates and predictors of HIV, hepatitis C virus (HCV), and hepatitis B virus (HBV) infection and therefore targeted young, recently initiated IDUs at risk for HCV infection. Participants were eligible if they were age 15 to 40 years and reported injecting drug use of heroin or cocaine at least once in the last two months but for no longer than 5 years. The NIDU study was also designed to investigate correlates and predictors of HIV, HCV and HBV and recruited young NIDUs. Participants were eligible if they were age 15 to 40 years and reported non-injecting drug use of heroin or cocaine at least once per week in the last two months but for no longer than 10 years, and no history of injecting drug use. All participants were reimbursed $20 for their participation. The study was approved by the institutional review board of the New York Academy of Medicine.

### Data collection

Following informed consent, eligible participants completed a standardized, detailed risk behavior questionnaire administered by trained interviewers. Participants were asked about sociodemographic characteristics, type and frequency of drug use, injection drug use practices, sexual behaviors and sexual partnerships. The absence of prior drug use by injection was confirmed during the comprehensive interviewing process and by phlebotomists' observations during venipuncture. Street methadone use was determined by asking, "Have you ever used street methadone (not from a program)?" and "During the last 6 months, how often did you use street methadone (not from a program)?"

Sociodemographic characteristics considered included age, race, gender, education, recent homelessness, main income source, and sexual identity. Main income source was divided into one of four categories: employed (full or part time employment, self-employed or temporary/off-books employment), illegal (selling drugs, sex for money, theft), public assistance (public assistance, welfare, social security benefits, or state or federal benefits including food stamps, state public aid, disability, or unemployment) and other (parent, friend, relative or spouse gave money; recycling cans; returning bottles for deposits; windshield wiping; or panhandling for money). Sexual identity was defined as heterosexual, men who have sex with men (MSM) and women who have sex with women (WSW). We examined recent frequency of substance use in the last six months, focusing on street (illicit) methadone, alcohol, marijuana, heroin, crack and cocaine.

Informed consent was obtained in writing from all participants.

### Statistical methods

We compared sociodemographic characteristics, drug use, sexual behaviors, and sexual partnerships between those who had used illicit methadone in the last six months and those who had not, stratified by injection status. Bivariable analyses were conducted to assess demographic and risk behavior variables by recent (last six month) illicit methadone use using chi-square statistics for categorical variables and *t *tests for continuous variables. Covariates that were significantly associated with recent illicit methadone use in univariable analyses (p < 0.10) were entered into a multivariable logistic regression model. Only those variables significant at p < 0.05 were retained in models. Separate models describing the correlates of street methadone use were constructed for injection and non-injection drug users.

## Results

Table 1 presents sociodemographic characteristics of the samples. Of 955 NIDUs, most were male (69.5%) and heterosexual (82.3%). Hispanics and Blacks were equally represented (45.0% and 45.7%, respectively). The mean age was 30.1 and more than half (57.5%) had recently been homeless. Of 460 IDUs, most were male (80.4%) and heterosexual (85.9%). The sample was majority Hispanic (81.1%); only 4.6% were Black. The mean age was 26.4 and most (72.2%) had recently been homeless.

**Table 1:** Socio-demographics of 1415 drug users, by illicit methadone (IM) use and injection status

	**Non-injection drug users**				**Injection drug users**			
	***Total***	***Current IM use***	***No current IM use***		***Total***	***Current IM use***	***No current IM use***	
	***n = 955***	***n = 113***	***n = 842***		***n = 460***	***n = 157***	***n = 303***	
	***n(%)***^*d*^	***n(%)***	***n(%)***	***p-value***^*a*^	***n(%)***^*d*^	***n(%)***	***n(%)***	***p-value***^*a*^
**Sex**								
*Male*	664 (69.5)	76 (67.3)	588 (69.8)	0.576	370 (80.4)	125 (79.6)	245 (80.9)	0.751
*Female*	289 (30.3)	37 (32.7)	252 (29.9)	0.541	88 (19.1)	30 (19.1)	58 (19.1)	0.993
*Transgender*	2 (0.2)	0 (0)	2 (0.2)	1.000^b^	2 (0.4)	2 (1.3)	0 (0)	0.120^b^
**Race**								
*Hispanic*	430 (45.0)	67 (59.3)	363 (43.1)	0.001	373 (81.1)	133 (84.7)	240 (79.2)	0.172
*Black*	436 (45.7)	36 (31.9)	400 (47.5)	0.002	21 (4.6)	5 (3.2)	16 (5.3)	0.304
*White*	25 (2.6)	1 (0.9)	24 (2.9)	0.960^b^	51 (11.1)	16 (10.2)	35 (11.6)	0.651
*Other*	64 (6.7)	9 (8.0)	55 (6.5)	0.567	14 (3.0)	3 (1.9)	11 (3.6)	0.398^b^
**Mean age (SD)**^c^	30.1 (6.5)	31.8 (6.2)	29.8 (6.5)	0.002	26.4 (5.3)	26.1 (4.9)	26.6 (5.5)	0.388
**Sexual orientation**								
*Heterosexual*	786 (82.3)	100 (88.5)	686 (81.5)	0.033	395 (85.9)	133 (84.7)	262 (86.5)	0.961
*MSM*^*e*^	90 (9.4)	5 (4.4)	85 (10.1)	0.051	13 (8.3)	32 (10.6)	13 (8.3)	0.447
*WSW*^*f*^	102 (10.7)	9 (8.0)	93 (11.0)	0.327	11 (7.0)	21 (6.9)	11 (7.0)	0.955
**< High school education**	526 (55.1)	68 (60.2)	458 (54.4)	0.246	190 (41.3)	67 (42.7)	123 (40.6)	0.709
**Homeless in last 6 months**	549 (57.5)	60 (53.1)	489 (58.1)	0.315	332 (72.2)	117 (74.5)	215 (71.0)	0.419
**Main source of income**								
Employed	180 (18.8)	18 (15.9)	162 (19.2)	0.334	59 (12.8)	20 (12.7)	39 (12.9)	0.925
*Illegal*	386 (40.4)	53 (46.9)	333 (39.5)	0.203	254 (55.2)	99 (63.1)	155 (51.2)	0.021
*Public Assistance*	221 (23.1)	27 (23.9)	194 (23.0)	0.956	65 (14.1)	16 (10.2)	49 (16.2)	0.071
*Other*	146 (15.3)	15 (13.3)	131 (15.6)	0.459	72 (15.7)	20 (12.7)	52 (17.2)	0.194
**Have children**	547 (57.3)	77 (68.1)	470 (55.8)	0.013	216 (47.0)	70 (44.6)	146 (48.2)	0.463
**Ever been incarcerated**	661 (69.2)	88 (77.9)	573 (68.1)	0.044	323 (70.2)	112 (71.3)	211 (69.6)	0.632
**Site**								
Bronx	348 (36.4)	43 (38.1)	305 (36.2)	0.704	232 (50.4)	83 (52.9)	149 (49.2)	0.453
*Harlem*	449 (47.0)	53 (46.9)	396 (47.0)	0.980	171 (37.2)	59 (37.6)	112 (37.0)	0.907
*Brooklyn*	67 (7.0)	11 (9.7)	56 (6.7)	0.228	22 (4.8)	6 (3.8)	16 (5.3)	0.487
*Queens*	47 (4.9)	5 (4.4)	42 (5.0)	0.880	6 (1.3)	3 (1.9)	3 (1.0)	0.415
*Lower East Side*	40 (4.2)	0 (0)	40 (4.8)	1.000^b^	27 (5.9)	6 (3.8)	21 (6.9)	0.178
**HIV+**	101 (10.6)	6 (5.3)	95 (11.3)	0.056	22 (4.8)	6 (3.8)	16 (5.3)	0.457
**HBV+**	211 (22.1)	24 (21.2)	187 (22.2)	0.832	141 (30.7)	47 (29.9)	94 (31.0)	0.763
***HCV+***	36 (3.8)	6 (5.3)	30 (3.6)	0.247^b^	255 (55.4)	86 (54.8)	169 (55.8)	*0.596*

a chi-square unless otherwise indicated

b Fisher's exact test

c ttest

d Column percents may not add up due to missing values

e MSM = men who have sex with men (by behavior or orientation); applies to males only

f WSW = women who have sex with women (by behavior or orientation); applies to females only

Approximately 21.8% of NIDUs had used illicit methadone (also referred to as "street methadone" in local parlance) in their lifetime (data not shown) and 11.8% had used within the last 6 months. In terms of frequency of use in the last six months, 5.8% used illicit methadone once or month or less, 1.5% used 2–3 days per month and 3.8% used at least once per week or more (data not shown). Only 0.9% used on a daily basis. The mean age of onset for any heroin use was 20.5 years while the age of onset for illicit methadone was 25.2 years. Of the 208 NIDU lifetime illicit methadone users 9 (4.3%) first used illicit methadone before they started using heroin, 31 (14.9%) started using illicit methadone and heroin at the same time, and 159 (76.4%) first used illicit methadone after they started using heroin. In bivariate analysis (table 1), NIDUs who had recently used illicit methadone were more likely than non users to be Hispanic [Odds Ratio (OR) = 1.9, 95% Confidence Interval (CI) = 1.3, 2.9], aged > 30 (OR = 1.5, 95% CI = 1.0, 2.2), heterosexual (OR = 2.1, 95% CI = 1.1, 4.0), have children (OR = 1.7, 95% CI = 1.1, 2.6), and been incarcerated (OR = 1.7, 95% CI = 1.0, 2.6). Methadone users were less likely than non users to be Black (OR = 0.5, 95% CI = 0.3, 0.8) and an MSM (OR = 0.4, 95% CI = 0.2, 1.0).

Approximately half (52.6%) of the IDU sample had used illicit methadone in their lifetime (data not shown) and 34.1% had used within the last 6 months. In terms of frequency of use in the last six months, 12.8% used illicit methadone once a month or less, 6.5% used 2–3 days per month and 11.3% used at least once per week or more (data not shown). Only 3.5% used on a daily basis. The mean age of onset for any heroin use was 19.4 years while the age of onset for illicit methadone was 23.1 years. Of the 242 IDU lifetime illicit methadone users 15 (6.2%) first used illicit methadone before they started using heroin, 31 (12.8%) started using illicit methadone and heroin at the same time, and 196 (81.0%) first used illicit methadone after they started using heroin. 165 (68.2%) initiated illicit methadone and injection at the same age.

There were no significant difference with respect to gender, race, age and sexual orientation among IDUs who had recently used illicit methadone compared to those who had not. However, illicit methadone users were less likely to have public assistance (OR = 0.6, 95% CI = 0.3, 1.1) and more likely to have an illegal source as their main income source (OR = 1.6, 95% CI = 1.1, 2.4).

In terms of substance use (table 2), NIDU illicit methadone users were more likely than non users to be polysubstance users (OR = 9.4, 95% CI = 2.3, 38.6), daily heroin users (OR = 5.1, 95% CI = 3.4, 7.8), less than daily methamphetamine users (OR = 3.5, 95% CI = 1.2, 10.3), heroin dependent (OR = 15.3, 95% CI = 8.3, 28.4), and cocaine dependent (OR = 1.5, 95% CI = 1.0, 2.2). They were also more likely to have experienced an overdose (OR = 2.1, 95% CI = 1.2, 3.6) and withdrawal symptoms (OR = 4.3, 95% CI = 2.8, 6.7) as compared to non users. NIDU illicit methadone users were also more likely to have been in a detoxification program (OR = 1.6, 95% CI = 1.0, 2.6) and methadone maintenance program (OR = 6.7, 95% CI = 2.9, 15.7) in the past six months compared to non users.

**Table 2:** Drug use and sexual behaviors of 1415 drug users by illicit methadone (IM) use and injection status

	**Non-injection drug users**				**Injection drug users**			
	***Total***	***Current IM use***	***No current IM use***		***Total***	***Current IM use***	***No current IM use***	
	***n = 955***	***n = 113***	***n = 842***		***n = 460***	***n = 157***	***n = 303***	
	***n(%)***^*d*^	***n(%)***	***n(%)***	***p-value***^*a*^	***n(%)***^*d*^	***n(%)***	***n(%)***	***p-value***^*a*^
**Lifetime drug use**								
*Polysubstance use*^*e*^	831 (87.0)	111 (98.2)	720 (85.5)	< 0.001	455 (98.9)	155 (98.7)	300 (99.1)	0.779^b^
*Crack*	722 (75.6)	91 (80.5)	631 (74.9)	0.194	383 (83.3)	136 (86.6)	247 (81.5)	0.164
*Cocaine*	902 (94.5)	107 (94.7)	795 (94.4)	0.906	451 (98.0)	154 (98.1)	297 (98.0)	0.959
*Heroin*	631 (66.1)	111 (98.2)	520 (61.8)	< 0.001	458 (99.6)	157 (100.0)	301 (99.3)	0.549^b^
**Current drug use (in last 6 months)**								
Crack								
*None*	226 (23.7)	22 (19.5)	204 (24.2)	0.264	84 (18.3)	21 (13.4)	63 (20.8)	0.051
*Less than daily*	374 (39.2)	50 (44.2)	324 (38.5)	0.238	189 (41.1)	72 (45.9)	117 (38.6)	0.134
*Daily*	236 (24.7)	25 (22.1)	211 (25.1)	0.497	69 (15.0)	25 (15.9)	44 (14.5)	0.690
Cocaine								
*None*	53 (5.5)	6 (5.3)	47 (5.6)	0.906	27 (5.9)	7 (4.5)	20 (6.6)	0.354
*Less than daily*	613 (64.2)	72 (63.7)	541 (64.3)	0.911	252 (54.8)	87 (55.4)	165 (54.5)	0.845
*Daily*	91 (9.5)	14 (12.4)	77 (9.1)	0.270	136 (29.6)	58 (36.9)	78 (25.7)	0.013
Heroin								
*None*	320 (33.5)	1 (0.9)	319 (37.9)	< 0.001	4 (0.9)	0 (0)	4 (1.3)	1.000^b^
*Less than daily*	354 (37.1)	59 (52.2)	295 (35.0)	< 0.001	190 (41.3)	48 (30.6)	142 (46.9)	0.001
*Daily*	167 (17.5)	51 (45.1)	116 (13.8)	< 0.001	303 (65.9)	131 (83.4)	172 (56.8)	< 0.001
Methamphetamine								
*None*	892 (93.4)	101 (89.4)	791 (93.9)	0.067	415 (90.2)	137 (87.3)	278 (91.7)	0.098
*Less than daily*	16 (1.7)	5 (4.4)	11 (1.3)	0.030^b^	12 (2.6)	7 (4.5)	5 (1.7)	0.073^b^
*Daily*	1 (0.1)	0 (0)	1 (0.1)	1.000^b^	3 (0.7)	0 (0)	3 (1.0)	1.000^b^
Marijuana								
*None*	163 (17.1)	19 (16.8)	144 (17.1)	0.922	104 (22.6)	23 (14.6)	81 (26.7)	0.003
*Less than daily*	342 (35.8)	47 (41.6)	295 (35.0)	0.184	187 (40.7)	72 (45.9)	115 (38.0)	0.114
*Daily*	381 (39.9)	40 (35.4)	341 (40.5)	0.281	148 (32.2)	56 (35.7)	92 (30.4)	0.268
Alcohol								
*None*	133 (13.9)	16 (14.2)	117 (13.9)	0.939	122 (26.5)	37 (23.6)	85 (28.1)	0.301
*Less than daily*	624 (65.3)	76 (67.3)	548 (65.1)	0.649	294 (63.9)	104 (66.2)	190 (62.7)	0.454
*Daily*	195 (20.4)	21 (18.6)	174 (20.7)	0.606	44 (9.6)	16 (10.2)	28 (9.2)	0.743
**Drug dependence, past year**								
*Heroin dependence*	381 (39.9)	97 (85.8)	284 (33.7)	< 0.001	421 (91.5)	156 (99.4)	265 (87.5)	< 0.001
*Crack dependence*	509 (53.3)	62 (54.9)	447 (53.1)	0.846	213 (46.3)	77 (49.0)	136 (44.9)	0.595
*Cocaine dependence*	516 (54.0)	70 (61.9)	446 (53.0)	0.069	308 (67.0)	105 (66.9)	203 (67.0)	0.674
*Withdrawal symptoms in last 6 months*	343 (35.9)	72 (63.7)	271 (32.3)	< 0.001	276 (60.0)	104 (66.2)	172 (56.8)	0.003
**Drug treatment, last 6 months**								
*Detoxification*	167 (17.5)	28 (24.8)	139 (16.5)	0.034	171 (37.2)	47 (29.9)	124 (40.9)	0.018
*Methadone maintenance*	145 (15.2)	35 (30.1)	110 (13.1)	< 0.001	167 (36.3)	120 (39.6)	47 (29.9)	0.942
**Medical outcomes**								
*HIV+*	101 (10.6)	6 (5.3)	95 (11.3)	0.056	22 (4.8)	6 (3.8)	16 (5.3)	0.457
*HCV+*	36 (3.8)	6 (5.3)	30 (3.6)	0.247^b^	255 (55.4)	86 (54.8)	169 (55.8)	0.596
*HBV+*	211 (22.1)	24 (21.2)	187 (22.2)	0.832	141 (30.7)	47 (29.9)	94 (31.0)	0.763
*Overdose*	94 (9.8)	19 (16.8)	75 (8.9)	0.008	96 (20.9)	39 (24.8)	57 (18.8)	0.131
**Sexual behaviors**								
*Mean age 1*^*st *^*intercourse (SD)*^*c*^	14.0 (3.0)	13.9 (3.1)	14.0 (2.9)	0.652	14.1 (2.7)	14.5 (2.4)	13.9 (2.8)	0.020
*Mean number of sexual partners (SD)*^*c*^	4.8 (14.2)	3.6 (7.5)	4.9 (14.8)	0.144	3.9 (9.8)	4.7 (13.6)	3.4 (6.7)	0.319
*Traded sex for money/drugs*	304 (31.8)	32 (28.3)	272 (32.3)	0.389	131 (28.5)	48 (30.6)	83 (27.4)	0.487
***Sexual partners in last 2 months***								
*Crack user*	354 (37.1)	38 (33.6)	316 (37.5)	0.655	96 (20.9)	32 (20.4)	64 (21.1)	0.818
*Cocaine user*	323 (33.8)	41 (36.3)	282 (33.5)	0.496	104 (22.6)	41 (26.1)	63 (20.8)	0.239
*Heroin use*	230 (24.1)	54 (47.8)	176 (20.9)	< 0.001	123 (26.7)	47 (29.9)	76 (25.1)	0.247
*Lifetime IDU*	81 (8.5)	16 (14.2)	65 (7.7)	0.034	122 (26.5)	39 (24.8)	83 (27.4)	0.576
*Recent IDU*	50 (5.2)	10 (8.8)	40 (4.8)	0.069	110 (23.9)	36 (22.9)	74 (24.4)	0.693
*Partner MSM*^*f*^	388 (40.6)	36 (31.9)	352 (41.8)	0.061	156 (33.9)	51 (32.5)	105 (34.7)	0.573
*Hepatitis infected*	34 (3.6)	8 (7.1)	26 (3.1)	0.040^b^	38 (8.3)	12 (7.6)	26 (8.6)	0.639
*HIV infected*	47 (4.9)	3 (2.7)	44 (5.2)	0.930^b^	9 (2.0)	4 (2.5)	5 (1.7)	0.385^b^

^a ^chi-square unless otherwise indicated, ^b ^Fisher's exact test, ^c ^ttest, ^d ^Column percents may not add up due to missing values, ^e ^used more than one drug out of the following in lifetime: heroin, crack, cocaine, methamphetamine, ^f ^MSM = men who have sex with men

IDU illicit methadone users were more likely to be daily cocaine (OR = 1.7, 95% CI = 1.1, 2.6) and heroin users (OR = 3.9, 95% CI = 2.4, 6.2), less than daily methamphetamine users (OR = 2.8, 95% CI = 0.9, 8.9) and heroin dependent users (OR = 1.6, 95% CI = 1.5, 1.7). They were also more likely to have experienced withdrawal symptoms (OR = 2.0, 95% CI = 1.3, 3.1) and less likely to have been in a detoxification program (OR = 0.6, 95% CI = 0.4, 0.9) as compared to non users.

We also examined injection behaviors among the IDU sample (Table 3). IDU illicit methadone users were significantly more likely to have injected in a shooting gallery (OR = 1.8, 95% CI = 1.2, 2.7), rented, borrowed or bought injection equipment at a shooting gallery (OR = 1.9, 95% CI = 1.1, 3.3), shared needles (OR 1.5, 95% CI = 1.0, 2.3), and shared other injection paraphernalia (i.e. cookers, cottons and rinse water) (OR = 1.6, 95% CI = 1.1, 2.5).

**Table 3:** Injection risk behaviors of 460 IDUs in New York City by recent illicit methadone use

		**Illicit methadone use**		
	***Total***	***Current use***	***No current use***	
	***n = 460***	***n = 157***	***n = 303***	
	***n(%)***^*d*^	***n(%)***	***n(%)***	***p-value***
Shot up in a shooting gallery	150 (32.6)	65 (41.4)	85 (28.1)	0.005
Used equipment at shooting gallery (rented/borrowed/bought)	94 (20.4)	47 (29.9)	47 (15.5)	0.027
Shared tourniquet	155 (33.7)	60 (38.2)	95 (31.4)	0.249
Shared cooker, cotton, or rinse water	292 (63.5)	111 (70.7)	181 (59.7)	0.021
Shared needles	193 (42.0)	76 (48.4)	117 (38.6)	0.033

With respect to sexual behaviors and partner characteristics, NIDU illicit methadone users were significantly more likely to have sexual partners that used heroin (OR = 3.5, 95% CI = 2.3, 5.3), were lifetime (OR = 1.9, 95% CI = 1.1, 3.5) and recent IDUs (OR = 1.9, 95% CI = 1.0, 4.0) and hepatitis infected (OR = 2.4, 95% CI = 1.1, 5.4). They were less likely to have an MSM sex partner (OR = 0.7, 95% CI = 0.4, 1.0). There were no significant differences with respect to sexual partner characteristics among IDU illicit methadone users as compared to non users; however, they had a significantly older mean age at first intercourse (14.5 years vs. 13.9 years, p = 0.02).

Tables 4 and 5 present the final multivariate logistic regression models for illicit methadone use among NIDUs and IDUs, respectively. Recent NIDU illicit methadone users were significantly more likely than non users to be heroin dependent [Adjusted OR (AOR) = 13.0], less than daily methamphetamine users (AOR = 5.4), and to have a heroin using sexual partner in the last six months (AOR = 2.3). Recent IDU illicit methadone users were significantly more likely than non users to be daily heroin users [Adjusted OR (AOR) = 3.7] and to share cookers, cotton or rinse water (AOR = 1.6) and significantly less likely to have been in a detoxification program (AOR = 0.6) and to not have used marijuana in the last six months (AOR = 0.5).

**Table 4:** Multivariate logistic regression model for recent illegal methadone use among 955 non-injection drug users

	**Crude OR**	**Adjusted OR**
	***(95% CI)***	***(95% CI)***
Heroin Dependence	15.3 (8.3, 28.4)	13.0 (6.8, 25.0)
Current user of methamphetamines: less than daily	3.5 (1.2, 10.3)	5.4 (1.4, 20.1)
Sexual partner used heroin in last 2 months	3.5 (2.3, 5.3)	2.3 (1.4, 3.6)

**Table 5:** Multivariate logistic regression model for recent illegal methadone use among 955 injection drug users

	**Crude OR**	**Adjusted OR**
***In the last 6 months...***	***(95% CI)***	***(95% CI)***
Daily heroin use	3.8 (2.4, 6.2)	3.7 (2.3, 6.1)
Detoxification program	0.6 (0.4, 0.9)	0.6 (0.4, 0.9)
Shared cookers, cotton or rinse water	1.6 (1.1, 2.5)	1.6 (1.0, 2.4)
No marijuana use	0.4 (0.3, 0.8)	0.5 (0.3, 0.9)

## Discussion

Illicit use of methadone was not uncommon in this population of street-recruited drug users in NYC. Approximately 21.8% of NIDUs and 52.6% of IDUs in this sample of street-recruited drug users had used illicit methadone in their lifetime and 11.8 and 34.1% had used within the last six months, respectively. A previous study had estimated a 21.3% past month prevalence for illicit methadone among Puerto Rican IDUs in East Harlem, New York [25]. In Australia, the lifetime and six month prevalence of injected methadone was 18.4% and 11.0%, respectively [6]. Our data did not distinguish between route of administration and the Australian study did not report on non-injected illicit methadone use, thus comparison between the estimates is limited. In Montréal, the illicit methadone prevalence was 42.1% among those who preferred heroin [5]. Although we did not ask about drug preference, frequency of use can serve as a proxy measure. Among the NIDUs and IDUs, approximately 38.7% of daily heroin users and 19.7% of less than daily heroin users also used illicit methadone. Differences in the prevalence of illicit methadone use among these studies are probably due, in part, to differences in sampling schemes and differences in measurement. However, methadone prescribing policies differ between countries and also play an important role in MMTP and illicit methadone availability.

Our study revealed that illicit methadone users engaged in riskier behavior than those who did not use street methadone, as evidenced by the higher prevalence of overdose experiences, polysubstance use and risky sex partners among NIDU illicit methadone users. Humeniuk and colleagues have suggested that methadone injectors are riskier than those who do not inject methadone, with methadone injectors in their population being more likely to have overdosed, used heroin by themselves and be a polysubstance user [6]. Further, we observed that IDUs who used street methadone reported riskier injection practices compared to IDUs who did not. Specifically, they were more likely to attend a shooting gallery and buy, rent or borrow injection equipment at a shooting gallery as well as share needles and other injection paraphernalia (i.e. cookers, cotton and rinse water). This is a novel finding. Hopwood and colleagues reported that methadone syrup injecting in New South Wales was not associated with sharing injection equipment, but it was associated with reuse of methadone injecting equipment [26].

Predictably, recent illicit methadone use was associated with heroin dependence among NIDUs and daily heroin use among IDUs. It is likely that dependence and daily use are measuring the same underlying construct of severity of use. Coupled with the data demonstrating relatively low frequency of use and later age of onset as compared to other both heroin and injection drug use, these findings suggest that illicit methadone use is likely not a primary drug of choice. This finding parallels that of Lauzon and colleagues in Montréal [5], who reported a later age of onset for illicit methadone use as compared to heroin, cocaine and a variety of other substances. The association between heroin dependence, daily heroin use and illicit methadone use suggests that methadone is used ancillary to heroin.

In the multivariate model for NIDUs, having a heroin using partner in the last two months was associated a more than two-fold increase in the likelihood of illicit methadone use. One possible explanation is that the sex partners are also drug use partners, pooling resources to purchase drugs. This would then suggest that having a heroin using sex partner may be related to more severe heroin use. Less than daily methamphetamine use was associated with a more than five-fold increase in the likelihood of illicit methadone use. Considering the low prevalence of methamphetamine use in this population, this association may be spurious. Little is known about methamphetamine use in NYC, although several recent studies have examined its use among gay and bisexual men [27-29]. These findings need further exploration.

Illicit methadone use among IDUs was associated with a significant increase in the likelihood of sharing injection paraphernalia such as cookers, cottons and rinse water, even after adjusting for daily heroin use, suggesting that street methadone use may be part of a larger profile of risky drug use behavior. Surprisingly, IDU illicit methadone users had a significantly lower likelihood of recent detoxification treatment compared to non users. One possible explanation is that detoxification may be perceived as ineffective or undesirable to chronic heroin users. Another explanation may be that IDUs are using street methadone to detox themselves.

As with any study, this study is subject to several limitations. The study sample was limited to users of heroin, crack and cocaine and specifically recent initiates to injection drug users and those who had never injected drugs. Other groups that may use illicit methadone including former and current pain patients, individuals who abuse prescription opioids, youth and other populations were not included. Correlates of illicit methadone use among those who are not heroin users and among those whose drug of choice is methadone are likely different. Further, the extent to which these findings are generalizable to other settings with different demographic profiles is unknown. Our population was relatively young and had few White drug users and Black injectors.

Availability and popularity of specific substances vary by region and the extent to which heroin is a primary drug of choice can vary widely [30,31]. Opioid analgesic sales per 100,000 population also vary widely by state [20]. Bourgois [32] has pointed out that methadone prescribing philosophies can differ between cities such that availability of methadone may be different between cities. We did not have data on route of administration of street methadone and therefore cannot look specifically at methadone injection. We do not think that injection is a common route of administration; Humeniuk et al. [6] reported that less than 20% in a study of heroin users in Australia reported lifetime methadone injection.

These data do not provide a complete picture of illicit methadone use and thus are limited. Two important questions remain unanswered. First, the reasons for illicit methadone use were not investigated. There are several possible reasons that individuals may use illicit methadone, including unavailability of heroin, underdosing in methadone programs and/or pain management, and experimentation prior to entry into a methadone program. In Australia, one study reported that 58% of methadone injectors preferred injection because it provided quicker relief of opiate withdrawal symptoms than drinking the syrup [26], suggesting that underdosing may be an important consideration. A study of U.S. methadone maintenance facilities found that approximately one-third provided dose under the recommended level [33]. Some proportion of illicit methadone use might then be attributed to attempts at self-medication either by supplementing MMTP, reducing dependence on heroin without the use of a formal treatment program, or preventing withdrawal symptoms when heroin is otherwise not available as has been suggested in earlier studies [7,34]. A recent study of patients in a German detoxification ward reported that approximately one-third reported using diverted opiods as an attempt at self-detoxification, when a dose of prescribed methadone had been missed, or as a transition before entering methadone maintenance treatment [35]. It has also been suggested that low availability of methadone treatment slots may be an important factor in the creation of an illicit street-level market for methadone [5]. Although bivariate analyses demonstrated a relationship between MMTP and illicit methadone use among NIDUs, this association was not significant in the multivariate analysis. Only 30.4% of illicit methadone users were recently in MMTP, suggesting that illicit methadone use may not simply be a result of underdosing of MMTP patients or personal diversion of medication. From these data, it is unclear if the remaining 67.6% of illicit methadone users are former MMTP patients. Second, the illicit methadone sources of participants in this study remain unknown. Several avenues for acquisition are likely available, including methadone available due to theft from hospitals or pharmacies, diversion from maintenance programs, or diversion from pain management prescriptions.

## Conclusion

Despite these limitations, this study suggests that persons using illicit methadone use are likely to be heavily risk prone (i.e., heavy users and those who report high risk behaviors). Further studies can clarify the mechanisms that may lead to more refined approaches to respond to this treatment problem. Additional qualitative and quantitative research is needed to understand the context of illicit methadone use and potential targets for intervention.

## List of abbreviations

AOR: adjusted odds ratio; CI: confidence interval; HBV: hepatitis B virus; HCV: hepatitis C virus; IDU: injection drug user; MMTP: methadone maintenance treatment programs; MSM: men who have sex with men; NIDU: non-injection drug user; NYC: New York City; OR: odds ratio; US: United States; WSW: women who have sex with women.

## Competing interests

The authors declare that they have no competing interests.

## Authors' contributions

DCO, CMF, DV and SG designed the parent study. DCO and DV conceived of and designed the current analysis, and drafted the manuscript. CC conducted the analysis, contributed to the analysis design, and reviewed the manuscript. CMF, SG, and VF helped to draft the manuscript. All authors read and approved the final manuscript.

## Pre-publication history

The pre-publication history for this paper can be accessed here:


